# Delay in reaching health facilities and its associated factors among mothers giving birth in South Gondar zone hospitals, Northwest Ethiopia, 2020: A facility-based cross-sectional study

**DOI:** 10.3389/fgwh.2023.916978

**Published:** 2023-03-20

**Authors:** Bekalu Getnet Kassa, Gebrehiwot Ayalew Tiruneh, Abayneh Aklilu Solomon

**Affiliations:** ^1^ Department of Midwifery, College of Health Science, Debre Tabor University, Debre Tabor, Ethiopia; ^2^ Department of Women and Family Health, School of Midwifery, College of Health Science, University of Gondar, Gondar, Ethiopia

**Keywords:** prevalence, delay in reaching health facility, Ethiopia, health sector, maternal mortality

## Abstract

**Background:**

Delays in reaching health facilities are one of three models identified as major contributors to maternal mortality and morbidity in developing countries, including Ethiopia. However, little is known about the prevalence and associated factors of delays in reaching healthcare facilities in Ethiopia, particularly in rural areas.

**Objective:**

The aim of this study was to assess the prevalence of delays in reaching health facilities and associated factors among mothers who gave birth in South Gondar zone hospitals, Northwest Ethiopia, 2020.

**Methods:**

A cross-sectional study design was used from 28 November to 25 December 2020, with 417 mothers who had recently given birth participating in the study, selected through a simple random sampling technique. Data were collected using a face-to-face interview with pretested structured questionnaires. The EpiData software (version 3.1) was used to record collected data and then exported to SPSS (version 23) for statistical analysis. Bivariable and multivariable analyses were conducted, and the odds ratio with 95% CI was used to identify factors associated with delays in reaching health facilities. The statistical significance was declared at *p* < .05.

**Results:**

The prevalence of delay in reaching health facilities among mothers who gave birth in South Gondar zone hospitals were 50.6%. Mothers who had no antenatal care (ANC) visits [adjusted odd ratio (AOR) = 3.16, 95% CI = 1.52, 6.56], an unplanned pregnancy (AOR = 1.78, 95% CI = 1.16, 2.72), and a distance from home to a health facility greater than 5 km (AOR = 1.69, 95% CI = 1.08, 2.65) were positively associated with delays in reaching health facilities.

**Conclusions:**

The prevalence of delays in reaching health facilities was higher in the study area. Women's empowerment through health education about ANC follow-up, choice of family planning methods, and creating accessibility to health facilities are essential measures to minimize delays in reaching health facilities.

## Introduction

Childbirth is an important event in a woman's life, but it is vulnerable to morbidity and mortality, particularly in developing nations like Ethiopia, where institutional delivery is still uncertain. Ethiopia has a high maternal mortality rate of 412 per 100,000 live births, with home delivery being one of the top reasons for death (1).

Delays in reaching health facilities refer to the time interval from deciding to seek emergency care to starting to receive the first health care service. The first delays happen at the family unit and community level and reflect the decision to seek care for pregnancy complications. The second delay refers to the delays in reaching health facilities that provide emergency obstetric care, which is the most common extreme in a rural area. The third delay refers to the delays that happen in receiving care after arrival at the health facilities (2, 3).

Studies have shown that mothers who have experienced delays in institutional deliveries have faced a number of health problems for both the mother and neonate, such as antepartum hemorrhage, premature rupture of membranes, postpartum hemorrhage, and uterine rupture. According to the Ethiopian demographic and health survey, obstetric fistula and maternal and neonatal complications are highly related to mothers who had delays in institutional deliveries (4, 5). In Ethiopia, maternal and neonatal healthcare services were underutilized. A large proportion of complications and deaths occur within the first 48 h of birth (6, 7). However, the majority are preventable, but delays during obstetric emergency care can significantly worsen the outcome of pregnancy (8).

Maternal healthcare utilization is critical for reducing maternal and neonatal mortality and raising awareness of pregnancy danger signs and the benefits of using institutional delivery services, but home delivery is still common, particularly in remote regions. The new Sustainable Development Goals 2030 set a target of less than 199 and 70 maternal mortality ratios per 100,000 live births globally and in Ethiopia, respectively (5, 9).

Several factors have been identified as barriers to early access to skilled care providers, especially in developing countries; these include poverty, distance, lack of information, poor-quality services, decision-making power, perceived inequality of care at health facilities, geographical inaccessibility, and cultural beliefs and practices (2, 3, 10, 11). Therefore, further studies need to be conducted to find out the main factors for delays in reaching health facilities. We investigated the magnitude and factors associated with delays in reaching health facilities among mothers who gave birth in South Gondar zone hospitals, Northwest Ethiopia, 2020.

## Methods and materials

### Study setting

The study was conducted in the South Gondar Zone, which is located in the central Amhara region of Ethiopia, in the northwestern part of the country. The area is approximately 668 km away from Addis Ababa and 103 km from Bahir Dar. It consists of 18 woreda and a population of 2,609,823 (1,304,911 females and 1,304,912 males) respectively. The study area contains eight government hospitals, 96 public health centers, 140 private clinics, and 403 health posts.^1^

### Study design and period

An institutional-based cross-sectional quantitative study was conducted from 28 November 2020 to 25 December 2020.

### Population

The source population was all mothers who gave birth in South Gondar zone hospitals, while all mothers who gave birth and were discharged to home after immediate postnatal follow-up in South Gondar zone hospitals during the study period were considered as the study population. All mothers who gave birth and registered in the delivery registration book at selected hospitals were included in the study, whereas mothers who were admitted before the onset of labor and lived in the study area for less than 6 months were excluded from the study.

### Sample size determination

The sample size was calculated using the single population proportion formula, and the required sample size for this study was determined using the following assumptions: desired precision (d) = 5%, confidence level = 95% (*Zα*/2 = ±1.96 value), and the prevalence of delay in reaching health facilities among mothers was 44.0% (12). Hence, the final calculated sample size with a 5% non-response rate was 417.

### Sampling procedures

**Figure 1:**
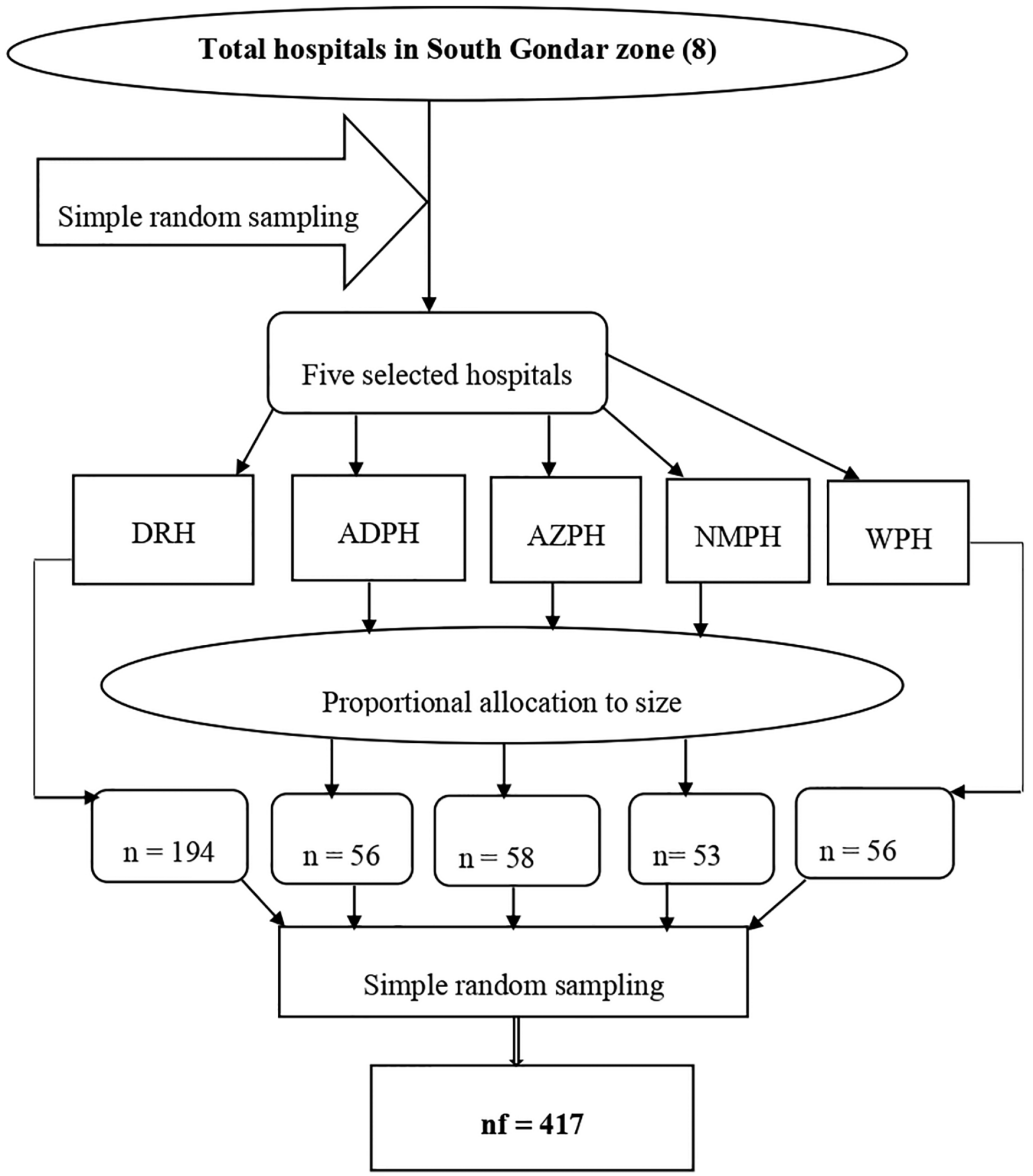
Schematic presentation of the sampling procedure among mothers in South Gondar zone hospitals, Northwest Ethiopia, 2020. DTGH, Debre Tabor Referral Hospital; AZPH, Addis Zemen Primary Hospital; NMPH, Nifas Mewucha Primary Hospital; WPH, Wogeda Primary Hospital.

In South Gondar zone, there are eight governmental hospitals; five out of eight hospitals were selected by simple random sampling methods to get a representative sample size. Then, a sample from each hospital was determined using proportional allocation according to size (PAS). Finally, simple random sampling was used to select all mothers who gave birth to achieve the desired sample size (Figure 1).

### Operational definitions

Delays to intuitional delivery refer to at least one or more delays from the three delays model (13).

Delay 2 (delay in reaching health facilities): refers to a mother unable to arrive within 1 h of traveling to reach the health facility by any mode transportation which is evidenced by the respondent's self-report (10, 14, 15).

### Study variables

#### Dependent variable

Delays in reaching health facility.

#### Independent variables

Sociodemographic characteristics (age, residence, marital status, ethnicity, religion, maternal and husband education, maternal and husband occupation, family income); obstetrics-related factors [gravidity, parity, antenatal care (ANC) follow up, type of pregnancy, past and current mode of delivery, knowing danger signs of labor] and health facility-related factors (available health facility, multiple referrals, distance of health facility, means of transportation, road accessibility, previous birthplace).

### Data collection procedures

The data collection tool was a structured questionnaire that was developed after a literature review, taking into account the local situation of the study area and the purpose of the study. First, the questionnaires were written in English, then translated into Amharic, the respondents' vernacular language, by a language expert for ease of comprehension. Face-to-face interviews were used to collect data.

### Quality assurances

Technical training was provided to data collectors prior to data collection, and pretesting was performed on 10% of the sample size. Data were collected by five trained diploma midwives, who were supervised by three bachelor's-degreed science midwives. Throughout the data collection period, the supervisors checked up on the process and double-checked each completed questionnaire for completeness. Finally, after entering the data, it was cleaned to ensure completeness.

### Statistical analysis

Data were coded, cleaned, and entered by Epidata version 3.1 and analyzed using computer database software and exported to the SPSS version 23 statistical software. Descriptive statistics, binary, and multivariable logistic regression analyses were used to identify factors associated with delay in reaching health facilities. Variables having *p*-value ≤.2 in the bivariable analysis were fitted into multiple logistic regression models to control the effect of confounding. The crude and adjusted odds ratio with their 95% CI were calculated to determine the strength and presence of association. A *p*-value of ≤.05 was considered to declare the level of significance.

## Results

### Sociodemographic characteristics

A total of 417 mothers were interviewed, with a response rate of 100%. Of the total study, 320 (76.7%) of the study participants' age was between 20 and 34 years, with a mean age of 27.23 years (SD ± 5.23 years). Almost all mothers, 415 (99.5%) were ethnic Amhara, and 403 (96.6%) were Orthodox Christian followers. Nearly two-thirds (62.7%) of mothers were urban residents. Furthermore, 209 (50.1%) of the study participants were government employees. As far as mothers' educational status is mentioned, at least 177 (42.4%) mothers had no formal education (Table 1).

**Table 1:** Sociodemographic characteristics of mothers who gave birth in South Gondar zone hospitals, Northwest Ethiopia, 2020.

Variables	Categories	Frequency	Percent
Age (years)	<20	39	9.4
	20–34	320	76.7
	≥35	58	13.9
Residence	Urban	261	62.6
	Rural	156	37.4
Marital status	Married	414	99.3
	Others*	3	0.7
Ethnicity	Amhara	415	99.5
	Others**	2	0.4
Religion	Orthodox	403	96.6
	Others***	16	3.3
Maternal education	No formal education	177	42.4
	Grade 1–8	74	17.7
	Grade 9–12	58	13.9
	College and above	108	25.9
Maternal occupation	Housewife	80	19.2
	Employed	209	50.1
	Merchant	45	10.8
	Student	19	4.6
	Farmer	41	9.8
	Daily labor	23	5.5
Husband education	No formal education	154	36.9
	Grade 1–8	76	18.2
	Grade 9–12	45	10.8
	College and above	142	34.1
Husband occupation	Employed	126	30.2
	Merchant	105	25.2
	Student	30	7.2
	Farmer	134	32.1
	Daily laborer	22	5.3

*Single, divorced, separated.

**Oromo, Tigrie, Guragie, Kimant.

***Muslim, Protestant, Catholic.

### Obstetrics-related factors characteristics

Regarding the types of pregnancy, 171 (41%) of mothers said that their pregnancy was planned. Three hundred fifty-six (85.4%) of mothers had a history of ANC visits during pregnancy. One-third (31.2%) of mothers had four or more ANC visits. As far as the pregnancy outcomes, 396 (94.9%) of mothers had a live birth in the last childbirth. Ninety (21.6%) of mothers were grand multipara. In terms of mode of delivery in the past, 176 (86.7%) of mothers gave birth by spontaneous vertex delivery. Nearly three-quarters (69.1%) of mothers decided to come to health facilities within 1 h of the onset of labor (Table 2).

**Table 2:** Obstetrics-related factors of mothers who gave birth in South Gondar zone hospitals, Northwest Ethiopia, 2020.

Variables	Categories	Frequency	Percent
Gravida	Primigravida	174	41.7
	Multigravida	153	36.7
	Grandmultigravida	90	21.6
Parity	1	170	40.8
	2–4	163	39.1
	≥5	84	20.1
ANC visits	Yes	356	85.4
	No	61	14.6
Number of ANC visits	No ANC	61	14.6
	1–3 visits	226	54.2
	4 and above	130	31.2
Planned pregnancy	Yes	171	41.0
	No	246	59.0
Pregnancy outcome	Livebirth	396	94.9
	Stillbirth	21	5.1
Birth weight of the baby	<2,500 g	47	11.3
	2,500–4,000 g	361	86.6
	≥4,000 g	9	2.2
Number of children	One	191	45.8
	2–4 children	139	33.3
	≥5 children	87	20.8
Mode of delivery in the past (*n* = 203)	SVD	176	86.7
	Instrumental delivery	11	5.4
	C/S	16	7.9
Current mode of delivery	SVD	348	83.5
	Instrumental delivery	28	6.7
	C/S	41	9.8
Complication of after delivery	Yes	35	8.4
	No	382	91.6
Knowledge of danger signs of labor	Yes	359	86.1
	No	58	13.9
Time of labor onset	Day	265	63.5
	Night	152	36.5
Decision to come to health institution after labor onset (delay 1)	<1 h	288	69.1
	≥1 h	129	30.9

C/S, caesarean section; SVD, spontaneous vertex delivery.

### Health facility-related variables

A total of 356 mothers (85.4%) said they had access to a health facility in a nearby kebele. More than half (54.7%) of mothers walked for less than an hour to get to a health facility. A total of 336 mothers (81.6%) said they were prepared to give birth at health facilities if labor began. Regarding referral status, more than one-third (34.8%) of mothers were sent to other health facilities for delivery services after being referred by another health facility. In terms of mode of transportation, 136 (32.6%) mothers went to the hospital by foot as their primary means of transportation (Table 3).

**Table 3:** Health facility factors of mothers who gave birth in South Gondar zone hospitals, Northwest Ethiopia, 2020 (*n* = 417).

Variables	Categories	Frequency	Percent
Presence of health facility in the Kebele	Yes	356	85.4
	No	61	14.9
Walking time from home to health facility	<1 h	228	54.7
	≥1 h	189	45.3
Readiness to deliver in the health institution	Yes	336	80.6
	No	81	19.4
Public transport service in your area go to the health facility	Yes	319	76.5
	No	98	23.5
How long wait to get service	<5 min	316	75.8
	≥5 min	101	24.2
The distance from home to health institution	<5 km	185	44.4
	≥5 km	232	55.6
Referred from other health facilities	Yes	145	34.8
	No	272	65.2
Mode of transportation	Foot	136	32.6
	Ambulance	128	30.7
	Private car	60	14.4
	Public transport	93	22.3

### Prevalence of delay in reaching health facilities

Among all study participants, the prevalence of delays in reaching health facilities was 50.6% (95% CI = 44.5%–55.2%).

### Factors associated with delay in reaching health facilities

Age, residence, maternal education, husband education, ANC visit, number of ANC visits, planned pregnancy, distance from home to health facility, receiving appropriate care, and being referred from one institution to another institution was found to be candidate variables for multivariable logistic regression analysis.

Multivariable logistic regression analysis revealed that ANC visits, planned pregnancies, and the distance from home to the health facility were significant predictors of delay in reaching the health facility at *p*-values .05.

Mothers who had no ANC visits were 3.158 times less likely to arrive at a health facility than mothers who had ANC visits [adjusted odd ratio (AOR) = 3.158, 95% CI = 1.520–6.563].

Mothers having an unplanned pregnancy are almost two times more likely to be prone to delays in reaching health facilities compared with their counterparts [AOR = 1.773, 95% CI = 1.157–2.717].

This study also demonstrated that travel time between home and the healthcare facility was a contributing factor to delays. Mothers whose traveling distance from home to a health facility was greater than 5 km experienced an almost 1.688-fold higher delay in reaching health facilities than those counterpart (AOR = 1.688, 95% CI = 1.075–2.650) (Table 4).

**Table 4:** Factors associated with delay in reaching health facility to institutional delivery of mothers who gave birth in South Gondar zone hospitals, Northwest Ethiopia, 2020.

Variables	Transportation delay		COR (95% CI)	AOR (95% CI)
	<60 min	≥60 min		
Age				
<20	18	21	0.671 (0.381–1.183)	0.910 (0.467–1.775)
20–34	164	156	0.824 (0.363–1.866)	1.204 (0.466–3.109)
≥35	24	34	1	1
Maternal education				
Unable to read and write	76	101	0.794 (0.461–1.369)	1.345 (0.722–2.539)
Grade 1–8	36	38	0.427 (0.231–0.788)	1.256 (0.636–2.484)
Grade 9–12	37	21	0.673 (0.416–1.089)	0.715 (0.356–1.437)
College and above	57	51	1	1
Residence				
Urban	135	126	1	1
Rural	71	85	1.283 (0.862–1.909)	0.766 (0.451–1.300)
ANC visit				
Yes	185	171	1	1
No	21	40	2.061 (1.168–3.635)	**3.158** (1.52–6.563)*
No of ANC visits				
No ANC	21	40	0.397 (0.211–0.747)	1.865 (0.152–3.019)
1–3 visit	111	115	0.544 (0.302–0.980)	2.21 (0.479–4.341)
Four and above	74	56	1	1
Planned pregnancy				
Yes	74	97	1	1
No	132	14	1.518 (1.54–3.39)	**1.773** (1.157–2.717)*
Distance from home to health facility				
<5 km	38	147	1	1
≥5 km	129	145	1.196 (1.067–5.284)	**1.688** (1.075–2.650)*
Receiving appropriate care				
Yes	162	154	1	1
No	44	57	1.363 (0.868–2.139)	1.353(0.854–2.142)

## Discussion

The findings revealed that 50.6% of mothers who gave birth in South Gondar zone hospitals experienced a delay in reaching health facilities. The result was consistent with the Zambian study (53.3%) (16). This could be due to similarities in the nature of the study, the study design, and the study population. Furthermore, it suggests a lack of the infrastructure needed for mothers to access health care facilities in an emergency.

On the other hand, this result was higher than the studies conducted in southwest Ethiopia (10, 13) and Myanmar (17), which found a delay was experienced by 43.2%, 29.7%, and 18% of mothers who visited delivery service facilities, respectively. This discrepancy might result from the different nature, design, and setting of the studies.

However, this finding is also lower than that of the study conducted in Malawi (18), in which 59.6% of mothers experienced a delay. Sociodemographic and cultural differences may explain this. In other perspectives, this difference may be due to the time gap between the study; as the study neared the present, participants' knowledge about obstetric complications improved, as did their attitudes toward delivery service utilization.

This study attempted to identify factors associated with delays in reaching health facilities in addition to estimating the prevalence. The multivariate analysis revealed that variables for delays in reaching the health facility, such as no ANC visits, unplanned pregnancy, and distance greater than 5 km, were statistically significant.

The World Health Organization (WHO) recommends that women without complications have at least four antenatal visits, one in each trimester (19). According to this study, mothers who did not receive antenatal care during their pregnancy were three times more likely to delay reaching a health facility than those who did receive antenatal care. This finding was similar to the studies conducted in the Arsi zone (14) and Kefa, Bench-Maji, and Sheka zones in south-west Ethiopia (20). This could be explained by the fact that ANC services can provide women with access to health information about their pregnancy status and the risk of giving birth at home, prompting them to make an informed decision about where to give birth. Furthermore, as explained by antenatal care, it is the best point of contact for mothers to learn more about the risks and complications that may arise during delivery.

Mothers who had an unplanned pregnancy were more likely to experience delays in reaching a health facility than mothers who had planned a pregnancy. This is supported by a study conducted in rural Ghana (21). In fact, women who have an unplanned pregnancy may be delayed in using prenatal care services, which may have an impact on the mother's and baby's health (22). These delays may be explained by the stigma of unplanned pregnancies and the perceived stigma from health professionals during delivery care due to cultural beliefs. Delayed disclosure of labor may contribute to unskilled assistance (such as relatives and traditional birth attendants) and is premised on the belief that women who inform the closest person immediately can delay the delivery. This, in turn, results in fear among mothers.

Distance is an important means of obtaining obstetric care for many pregnant and postpartum women in low-resource settings, particularly in rural areas (23). Many pregnant women do not even attempt to reach a delivery facility because walking long distances during labor is difficult and impossible if labor begins at night, and transportation is frequently unavailable. According to this finding, mothers who traveled more than 5 km from their home to a health facility were 1.69 times more likely to experience delays than their counterparts. This is supported by the study done southern in Ethiopia (10).

### Limitations

This analysis has two limitations. First, the research findings are cross-sectional, which limits the ability to assess the causal path between independent and dependent variables. Second, and most importantly, survey participants may under-report delays in reaching a health facility due to the stigma associated with traditional beliefs and cultural influence. Future research should overcome these limitations by incorporating quantitative and qualitative studies.

## Conclusions

The magnitude of the delay in reaching a health facility in the study area was high. Having no ANC visits, number of ANC visits, unplanned pregnancy, and a traveling distance from home to a health facility that is greater than 5 km were significantly associated with delay in reaching health facility. Awareness creation about antenatal visits, choice of family planning methods, and increasing infrastructure regarding improving institutional delivery are essential measures to minimize delays in reaching health facilities.

## Data Availability

The raw data supporting the conclusions of this article will be made available by the authors, without undue reservation.

## Footnotes

^1^Unpublished South Gondar zonal health office report (2019).

## References

[1] Central Statistics Agency I. Ethiopia Demographic and Health Survey 2016: Key Indicators Report. Addis Ababa, Ethiopia and Rockville, Maryland: CSA and ICF (2016). p. 659–65.

[2] Berhan Y Berhan A. Commentary: reasons for persistently high maternal and perinatal mortalities in Ethiopia: part III—perspective of the “three delays” model. Ethiop J Health Sci. (2014) 24:137–48. 10.4314/ejhs.v24i0.12S 25489188 PMC4249213

[3] Thaddeus S Maine D. Too far to walk: maternal mortality in context. Soc Sci Med. (1994) 38(8):1091–110. 10.1016/0277-9536(94)90226-7 8042057

[4] Asare EAM. (2017) Determinants of utilisation of maternal health care services among pregnant women in Kwahu South District. [MPhil thesis] [Accra, Ghana]: University of Ghana.

[5] Gabrysch S Campbell OM. Still too far to walk: literature review of the determinants of delivery service use. BMC Pregnancy Childbirth. (2009) 9(1):34. 10.1186/1471-2393-9-34 19671156 PMC2744662

[6] Debelew GT Afework MF Yalew AW. Factors affecting birth preparedness and complication readiness in Jimma Zone, Southwest Ethiopia: a multilevel analysis. Pan Afr Med J. (2014) 19:272. 10.11604/pamj.2014.19.272.4244 25870727 PMC4391899

[7] Central Statistics Agency (Ethiopia), Ministry of Health (Ethiopia), World Bank. Ethiopia Mini Demographic and Health Survey 2014. (2014) Addis Ababa, Ethiopia.

[8] Rodrigo CH Kumarapeli V. Birth preparedness, complication readiness and associated factors among pregnant women seeking antenatal care at a Medical Officer of Health (MOH) area in Sri Lanka. J Postgrad Med Inst. (2019) 6(1):1–12. 10.4038/jpgim.8194

[9] Institute of Ethiopia. National technical guidance for maternal and perinatal death surveillance and response, (2017).

[10] Lire A Beyamo A Tadele D Facha W. Delays for utilizing institutional delivery and associated factors among mothers attending public health facility in Hadiya Zone, Southern Ethiopia. Science. (2017) 5(6):149–57. 10.11648/j.sjph.20170506.13

[11] Killewo J Anwar I Bashir I Yunus M Chakraborty J. Perceived delay in healthcare-seeking for episodes of serious illness and its implications for safe motherhood interventions in rural Bangladesh. J Health Popul Nutr. (2006) 24(4):403. 17591337 PMC3001144

[12] Wanaka S Hussen S Alagaw A Tolosie K Boti N. Maternal delays for institutional delivery and associated factors among postnatal mothers at public health facilities of Gamo zone, Southern Ethiopia. Int J Womens Health. (2020) 2020:127–38. 10.2147/IJWH.S240608 32184676 PMC7061422

[13] Yarinbab TE Balcha SG. Delays in utilization of institutional delivery service and its determinants in Yem Special Woreda, Southwest Ethiopia: health institution based cross-sectional study. J Gynecol Women's Health. (2018) 10(3):555793.

[14] Amare YW Dibaba B Bayu M Hussien M. Factors associated with maternal delays in utilising emergency obstetric care in Arsi zone, Ethiopia. S Afr J Obstet Gynaecol. (2019) 25(2):56–63. 10.7196/sajog.1437

[15] Nahar S Banu M Nasreen HE. Women-focused development intervention reduces delays in accessing emergency obstetric care in urban slums in Bangladesh: a cross-sectional study. BMC Pregnancy Childbirth. (2011) 11(1):11. 10.1186/1471-2393-11-11 21276263 PMC3045375

[16] Gabrysch S Cousens S Cox J Campbell OM. The influence of distance and level of care on delivery place in rural Zambia: a study of linked national data in a geographic information system. PLoS Med. (2011) 8(1):e1000394. 10.1371/journal.pmed.1000394 21283606 PMC3026699

[17] Win T Vapattanawong P Vong-ek P. Three delays related to maternal mortality in Myanmar: a case study from maternal death review, 2013. J Health Res. (2015) 29(3):179–87. 10.14456/jhr.2015.4

[18] Mgawadere F Unkels R Kazembe A van den Broek N. Factors associated with maternal mortality in Malawi: application of the three delays model. BMC Pregnancy Childbirth. (2017) 17(1):1–9. 10.1186/s12884-017-1406-5 28697794 PMC5506640

[19] Yebyo H Alemayehu M Kahsay A. Why do women deliver at home? Multilevel modeling of Ethiopian National Demographic and Health Survey data. PLoS One. (2015) 10(4):e0124718. 10.1371/journal.pone.0124718 25874886 PMC4398378

[20] Yemaneh Y Tiruneh F. Proportion and Associated factors of maternal near misses in selected public health institutions of Keffa, Bench-Maji and Sheka zones of south nations nationalities and people regional state, south west Ethiopia, 2017: A cross-sectional study. (2018). 10.20944/preprints201804.0368.v1

[21] Sumankuuro J Mahama MY Crockett J Wang S Young J. Narratives on why pregnant women delay seeking maternal health care during delivery and obstetric complications in rural Ghana. BMC Pregnancy Childbirth. (2019) 19(1):260. 10.1186/s12884-019-2414-4 31337348 PMC6651920

[22] Moges Y Worku SA Niguse A Kelkay B. Factors associated with the unplanned pregnancy at Suhul General Hospital, Northern Ethiopia, 2018. J Pregnancy. (2020) 2020:2926097. 10.1155/2020/2926097 32685212 PMC7336205

[23] Okeshola FB Sadiq IT. Determinants of Home Delivery among Hausa in Kaduna South Local Government Area of Kaduna State, Nigeria. Am. Int. J. Contemp. Res. (2013) 3(5):78–85.

